# Research on risk factors of ischemic cerebrovascular disease in postmenopausal women based on the social-ecological model

**DOI:** 10.1186/s40001-022-00734-8

**Published:** 2022-07-04

**Authors:** Chun-Jun Yang, Dong-Mei Wang, Tong Wang, Ying Song

**Affiliations:** 1 Department of stomatology, Tianjin Medical University General Hospital, Tianjin, 300052 China; 2 Department of Healthcare, Tianjin Medical University General Hospital, 154 Anshan Road, Heping District, Tianjin, 300052 China

**Keywords:** Postmenopausal women, Ischemic cardiovascular disease, Social ecological model

## Abstract

**Objective:**

Based on the social-ecological model, this study aimed to comprehensively explore factors affecting the risk of ischemic cerebrovascular disease (ICVD) in postmenopausal women to provide theoretical bases for further prevention and intervention for postmenopausal women.

**Methods:**

Postmenopausal women who underwent medical examinations in one health-checkup agency in Tianjin from May 2015 to October 2015 were enrolled in this study. The ICVD 10-year Risk Assesment Form developed by the research team of the National "Tenth Five-Year Plan" research project was used to assess the factors affecting the risk of ICVD. Based on the social-ecological model, multiple types of scales, including physical activities, depression, Type D personality, social supports, and environment score, were used to comprehensively explore the factors associated with ICVD in postmenopausal women.

**Results:**

300 valid questionnaires were obtained, with an effective rate of 92.0%. The subjects aged 44–74 years, with the average age of 62.06 ± 7.09 years. Among them, 58.67% of the subjects only obtained high-school diploma, 32.67% obtained college or university diploma, 90.33% were retirees, 95.33% were married, 92.33% experienced the natural menopause, 93.33% lived in urban or suburban areas, and 1.00% had a history of breast cancer. Multivariate logistic regression analysis suggested that monthly income (¥), parity, exposure to second-hand or third-hand smoke, easy access to healthy food, physical activities, depression, Type D personality, social support and environmental factors were associated with the risk of ICVD in postmenopausal women (*P* < 0.05). Among them, easy access to healthy food (OR = 0.242), social support (OR = 0.861) and environmental factors (OR = 0.866) were protective factors from ICVD. OR < 1 indicates that the exposure factor is negatively correlated with the disease, and the exposure factor has a protective effect on preventing the occurrence of the disease. Parity (OR = 3.795), exposure to second-hand or third-hand smoke (OR = 2.886), depression (OR = 1.193), and Type D personality (OR = 1.148) were risk factors of ICVD. OR > 1 means that the exposure factor is positively correlated with the disease, and the exposure factor increases the risk of disease occurrence.

**Conclusions:**

For postmenopausal women, in the future, in addition to prevention and management of the conventional risks, the conditions of their mentality and social support should be paid attention to, at the same time, and if they can, try to choose a good community environment to live in, which could better reduce the incidence and mortality of ICVD in postmenopausal women.

## Introduction

With the development of economy and society, people’s diets and lifestyles have changed accordingly, especially the high-fat, high-salt and high-sugar diet and sedentary lifestyle, resulting in the increased incidence and mortality of cardiovascular diseases year by year [1–4]. For postmenopausal women, a lack of estrogen that has a protective effect on the cardiovascular system is associated with a significantly increased risk of cardiovascular disease [5–10]. Therefore, it has become a crucial problem in today’s society to explore the factors affecting the risk of cardiovascular disease in postmenopausal women. Social ecosystem theory (SET) is a new theoretical model based on the relationship between human behavior and social environment, which consists of three levels: micro-level system of individuals, meso-level system of family, work world or other social groups, and macro-level system of environment, community, etc., and stresses the comprehensive influences of individual factors, natural environment and social environment on human health [11–13]. However, currently, researches in our country mostly focus on sociodemographic characteristics and laboratory testing at the micro-level, and ignore the psychological factors at the micro-level, the social supports at the meso-level, and the community environment at the macro-level, which also affects the incidence of cardiovascular disease. The research team from the national "Tenth Five-Year Plan" research project of "Coronary Heart Disease and Stroke Comprehensive Risk Assessment and Intervention Program Research" takes into account that our country, as a developing country, is a country with relatively low incidence of coronary heart disease and relatively high incidence of stroke, and if the coronary heart disease risk assessment model from the western country is directly used to predict the risk of cardiovascular disease in the Chinese population, the prediction results may be inaccurate, and thus this research team used the follow-up data of 17,329 subjects to build a predictive model, and named the combined endpoint of coronary heart disease and ischemic stroke as ischemic cardiovascular disease (ICVD) [14]. Our present study, based on SET, aimed to explore the risk factors of ICVD in postmenopausal women from the above three levels, including micro-, meso-, and macro-level. It is expected to provide a theoretical basis for prevention and intervention of ICVD in postmenopausal women in the future.

## Materials and methods

This study was conducted in accordance with the declaration of Helsinki. This study was conducted with approval from the Ethics Committee of Tianjin Medical University General Hospital. Written informed consent was obtained from the participants.

### Subjects

Postmenopausal women who underwent medical examinations in one health-checkup agency in Tianjin from May 2015 to October 2015 were enrolled in this study.

Inclusion criteria: (1) patients had menopause for more than 1 year; (2) patients did not have medical history of cardiovascular disease, such as coronary heart disease, ischemic stroke, etc.; (3) patients was able to complete the questionnaire independently.

Exclusion criteria: (1) patients were unable to participate in daily physical activity; (2) patients had a medical history of psychosis; (3) patients were unwilling to participate in this study; (4) patients did not complete the questionnaire for various reasons.

### Calculation of sample size

According to the requirements of logistic regression analysis, the sample size is generally 20 to 30 times the number of variables. The number of variables in our present study was 12. Considering the drop out of the samples and the invalid questionnaire, we set the drop-out rate as 10% to obtain the sample size greater than 264. Thus, we finally determined to include 300 cases in this study.

### Theoretical framework

SET is designed to explore the relationship between the social ecological environment and human behavior, and how they interact and influence each other. This theory focuses on individuals in the social ecological system, and believes that interaction and mutual-influence of individuals and the environment are dynamic and coadapted, emphasizes that the social ecological environment is of great significance for the exploration and interpretation of human behavior, and to a certain extent, provides a more comprehensive theoretical basis for social work.

This theoretical model consists of micro-level system, meso-level system and macro-level system. The micro-level system refers to individuals who exist independently in the social ecological environment, with physical, psychological, and social attributes. The meso-level system refers to small-scale organizations that have an impact on individuals, including families, colleagues, or other social groups. The macro-level system refers to a social system that is larger than the meso-level system, mainly including environment, culture, community, etc. These three-level systems are always interacting and influencing each other. Therefore, in order to understand and solve problems more comprehensively, we should explore the underlying causes of human behavior from multiple levels and perspectives.

### Research tools

#### Basic questionnaire on general data

A self-designed basic questionnaire for general information, such as age, working condition, education, marriage status, body mass index (BMI), medical history, blood pressure, total cholesterol and triglycerides, was used.

#### Ischemic Cardiovascular Disease (ICVD) 10-year Risk Assessment Form

The "Ischemia Cardiovascular Disease (ICVD) 10-year Risk Assessment Form" [14] developed by the research team of the national "Tenth Five-Year Plan" research project was used to do the risk assessment of ICVD in postmenopausal women, including 6 risk factors: age, systolic blood pressure, total cholesterol, body mass index (BMI), diabetes mellitus (DM) and smoking. Each risk factor was given different risk scores according to different conditions of cases, and finally the sum of risk scores of 6 factors was calculated for each case. According to the absolute risk, the samples were divided into two groups: the low-risk group (absolute risk < 10%) and the high-risk group (absolute risk ≥ 10%).

#### Self-rating Depression Scale

The Self-rating Depression Scale (SDS) developed by W.K.Zung in 1965 [15]was used to assess the subjective feelings, including 20 items, 10 of which were scored in reverse. Reverse scoring question numbers were 2, 5, 6, 11, 12, 14, 16, 17, 18, and 20.

A 4-level scoring of 1, 2, 3, and 4 points was used, of which 1 point represents “no or very little time”, 2 points represent “a small amount of time”, 3 points represent “a lot of time”, and 4 points represent “most or all of the time”. Finally, the sum of the scores of 20 items was calculated to obtain the total rough score. The standard score = the total rough score × 1.25. According to the results of the Chinese norm, the cut-off value of the SDS standard score was 53 points, 53–62 points of standard score represents mild depression, 63–72 points of standard score represents moderate depression, 72 points of standard score represents severe depression, and the higher the standard score, the more severe the depression.

#### D-type Personality Scale

The D-type Personality Scale (DS14) with good reliability and validity [16] was developed by Denollet J, a Dutch scholar, and translated and revised by Yu XN and Zhang JX from the Chinese Academy of Sciences. Type D personality is defined as the presence of two personality characters, namely negative affectivity (NA) and social inhibition (SI), which is associated with various disorders. SI refers to people's repression of their own expressions of emotion and behavior in social interactions. Because they feel nervous and insecure when they are in contact with others, they consciously maintain a state of self-repression. NA refers to people who experience negative emotions over a long period of time, and such experiences tend to be relatively stable, independent of time and situation. They tend to be apprehensive and have pessimistic views about life. At the same time, they are more prone to anger than the average person, and are less likely to experience positive emotions.

The questionnaire DS14 has a total of 14 questions, including 7 questions of NA, and 7 questions of SI. A 5-level scoring of 0, 1, 2, 3, and 4 points was used to calculate each question, and 2 questions were scored in reverse. 0 point represents “completely inconsistent”, 1 point represents “less consistent”, 2 points represent “uncertain”, 3 points represent “somewhat consistent”, and 4 points represent “fully consistent”. The score of one dimension was the sum of the scores of the items in this dimension. The total score of the scale was the sum of the scores of all items in this scale. The SI score and the NA score were both greater than 10 points at the same time, which can be diagnosed as Type D personality. Higher scores indicate more severe Type D personality.

#### Social Support Rating Scale

Social Support Rating Scale (SSRS) with good reliability and validity [17] was developed led by Xiao SY in 1986. Cronbach 'a of the the total score is 0.92, and Cronbach’s a of each item score is 0.89–0.94. The scale has 10 items and consists of 3 dimensions, including objective support, subjective support and the rate of social support utilization. The score of one dimension is the sum of the scores of the items in this dimension, and the total score of social support is the sum of the scores of the three dimensions. The higher the total score is, the more social support is and the less social isolation is.

#### Abbreviated-Neighborhood Environment Walkability Scale

The Abbreviated-Neighborhood Environment Walkability Scale (ANEWS) [18] amended by Zhou Rena from the Fudan university in 2011 consists of 5 dimensions, including 17 items. The questionnaire uses a 5-level scoring from 1 to 5 scores. The score of one dimension is the sum of scores of items in this dimension, and the total score of the scale is the sum of the scores of all items. The higher the total score is, the better the walking environment is. Cronbach’s α of the scale is 0.807, the intra-group correlation coefficient (ICC) is 0.945, and the Spearman correlation coefficient is 0.721.

#### Women’s Health Initiative Physical Activity Questionnaire

The Women’s Health Initiative Physical Activity Questionnaire (WHI-PAQ) [19] developed by Anne-Marie Meyer, a American scholar, and translated and revised by Yang CJ, mainly consists of two parts, including physical exercise and housework, with a total of 8 items, such as walking, light, moderate and heavy physical exercise, housework, garden activities, and sitting and sleeping time. The formula is: total energy consumption = (weekly activity frequency × minutes per time × corresponding metabolic equivalent) / (60 min/hour). WHI-PAQ classifies the intensity of exercise into 3 levels according to total energy consumption, including low-intensity exercise (< 500 MET × min × wk (−1)), medium-intensity exercise (500–1200 MET × min × wk (−1)) and high-intensity exercise (> 1200 MET × min × wk (−1)). The test–retest reliability of the scale is 0.950, and each dimension is 0.957–0.669. The Spearman rank correlation coefficient of overall standard validity is 0.884, the content validity index (CVI) is 0.923, and the content validity ratio (CVR) is 0.846, with good reliability and validity.

## Methods for performing the questionnaire survey

The researcher conducted a questionnaire survey for postmenopausal women who met the inclusion criteria. Before filling out the questionnaire, based on the guidelines, how to fill out and what to note was introduced by the researchers. After completing the questionnaires, the researchers checked the submitted questionnaires on the spot. For the questionnaires with the missing items, remedial measures were taken in time, and then the researchers took them back without any missing items after checking again.

## Statistical analysis

SPSS 18.0 statistical software was used for data entry and processing, and double entry and validation were used. If the quantitative data conformed to the normal distribution, the mean ± standard deviation (SD) was used, and the difference between groups was analyzed by t-test. If quantitative data did not conform to a normal distribution, the median and inter-quartile range were used to describe the data, and the difference between groups was analyzed by rank-sum test. For qualitative data, frequency and percentage were used to describe the data, and the difference between groups was analyzed by Chi-square test. Binary logistic regression analysis was used to analyze and explore the factors affecting the occurrence of ICVD in postmenopausal women, and *P* < 0.05 in two sides was considered statistically significant.

## Results

### Socio-demographic data of the enrolled postmenopausal women

A total of 326 questionnaires were collected. After the questionnaires with incomplete data were removed, 300 valid questionnaires were obtained, with an effective rate of 92.0%. The maximum age of the subjects was 74 years, the minimum age was 44 years, and the average age was 62.06 ± 7.09 years. Among them, 58.67% of the subjects only obtained high-school diploma, 32.67% obtained college or university diploma, 90.33% were retirees, 95.33% were married, 92.33% experienced the natural menopause, 93.33% lived in urban or suburban areas, and 1.00% had a history of breast cancer.

### Statistical analysis of factors of ICVD in postmenopausal women

#### Comparison of factors related to ICVD in postmenopausal women between low- and high-risk groups using independent sample t test for numerical variables

Independent sample t-test analysis indicated that there were significant differences in monthly income, gravidity, parity, physical activities, depression, Type D personality, social supports and environment score between the low-risk group and the high-risk group (*P* < 0.05) (Table 1).

**Table 1:** Comparison of factors related to ischemic cerebrovascular disease in postmenopausal women between low- and high-risk groups using independent sample *t* test for numerical variables

Factors	Low-risk group (n = 207) $\overline{x} \pm s$	High-risk group (n = 93) $\overline{x} \pm s$	*t*	*P*
TG (mmol/L)	1.63 ± 1.31	1.74 ± 1.40	−0.68	0.505
Monthly income (¥)	2931.40 ± 859.91	3230.75 ± 1004.59	−2.65	0.009**
Age of menarche (years old)	14.4 ± 2.0	14.8 ± 1.8	−1.74	0.083
Age of first delivery (years old)	27.4 ± 3.1	26.7 ± 2.4	1.77	0.077
Age of menopause (years old)	49.8 ± 3.7	50.6 ± 4.5	−1.67	0.095
Parity (times)	1.56 ± 0.84	1.96 ± 0.82	−3.61	< 0.001***
Gravidity (times)	1.19 ± 0.49	1.75 ± 0.71	−7.93	< 0.001***
Physical activity (scores)	4268.43 ± 1681.15	2325.43 ± 939.13	10.43	< 0.001***
Depression (scores)	41.05 ± 5.88	50.48 ± 6.06	−12.72	< 0.001***
Type D personality (scores)	11.52 ± 6.19	19.03 ± 6.43	−9.61	< 0.001***
Social support	35.34 ± 6.38	27.65 ± 4.49	10.51	< 0.001***
Environment (scores)	58.44 ± 9.29	53.35 ± 4.75	5.09	< 0.001***

^*^P < 0.05; **P < 0.01;***P < *0.001*

#### Comparison of factors related to ICVD in postmenopausal women between low- and high-risk groups using Chi-square test for categorical variables

The *χ*^*2*^ test analysis suggested there were significant differences in working conditions, whether to be exposed to second-hand or third-hand smoke, whether to easily buy healthy food, and whether to participate in lectures on cardiovascular disease between the low-risk group and the high-risk group (*P* < 0.05) (Table 2).

**Table 2:** Comparison of factors related to ischemic cerebrovascular disease in postmenopausal women between low- and high-risk groups using Chi-square test for categorical variables

Factors		Low-risk group (*n* = 207)	High-risk group (*n* = 93)	*χ*^*2*^	*P*
Working conditions	On job (*n*, %)	26 (12.6%)	1 (1.1%)	14.23	0.001**
	Retirement (*n*, %)	180 (86.9%)	91 (97.8%)		
	Without job (*n*, %)	1 (0.5%)	1 (1.1%)		
Education	Primary school or without (*n*, %)	8 (3.9%)	2 (2.1%)	3.06	0.383
	High school (*n*, %)	120 (57.9%)	56 (60.2%)		
	College or university (*n*, %)	79 (38.2%)	34 (36.6%)		
	Graduate school (*n*, %)	0 (0.0%)	1 (1.1%)		
Marriage status	Being married (*n*, %)	198 (95.7%)	88 (94.6%)	4.54	0.104
	Being widowed (*n*, %)	5 (2.4%)	5 (5.4%)		
	Being divorced (*n*, %)	4 (1.9%)	0 (0.0%)		
Ways of menopause	Natural (*n*, %)	189 (91.3%)	88 (94.6%)	1.06	0.303
	Surgical (*n*, %)	18 (8.7%)	5 (5.4%)		
Types of health care	Basic health insurance (*n*, %)	197 (95.2%)	89 (95.7%)	6.21	0.184
	The rural cooperative medical care (*n*, %)	3 (1.4%)	0 (0.0%)		
	Out-of-pocket medical services (*n*, %)	2 (1.0%)	0 (0.0%)		
	Medical services at state expense (*n*, %)	5 (2.4%)	3 (3.2%)		
	Commercial health insurance (*n*, %)	0 (0.0%)	1 (1.1%)		
Residence	Rural (*n*, %)	4 (1.9%)	0 (0.0%)	3.33	0.189
	Urban (*n*, %)	12 (5.8%)	4 (4.3%)		
	Suburban (*n*, %)	191 (92.3%)	89 (95.7%)		
Drinking	Yes (*n*, %)	11 (5.3%)	1 (1.1%)	3.74	0.053
	No (*n*, %)	196 (94.7%)	92 (98.9%)		
Salt intake per day	3–6 g (*n*, %)	145 (70.0%)	68 (73.1%)	0.30	0.586
	> 6 g (*n*, %)	62 (30.0%)	25 (26.9%)		
Oil intake per day	30-50 g (*n*, %)	156 (75.4%)	75 (80.6%)	1.04	0.309
	> 50 g (*n*, %)	51 (24.6%)	18 (19.4%)		
Dietary habit	Balance of meat and vegetables (*n*, %)	133 (64.2%)	58 (62.4%)	2.70	0.259
	Most of meat (*n*, %)	8 (3.9%)	8 (8.6%)		
	Most of vegetables (*n*, %)	66 (31.9%)	27 (29.0%)		
Breast cancer	Yes (*n*, %)	1 (0.5%)	2 (2.2%)	1.63	0.202
	No (*n*, %)	206 (99.5%)	91 (97.8%)		
Taking hormones	Yes (*n*, %)	4 (1.9%)	4 (4.3%)	1.29	0.257
	No (*n*, %)	203 (98.1%)	89 (95.7%)		
House facing the street	Yes (*n*, %)	53 (25.6%)	19 (20.4%)	0.96	0.327
	No (*n*, %)	154 (74.4%)	74 (79.6%)		
Second-hand or third-hand smoke	Yes (*n*, %)	80 (38.6%)	22 (23.7%)	6.67	0.010*
	No (*n*, %)	127 (61.4%)	71 (76.3%)		
Easy access to healthy food	Yes (*n*, %)	195 (94.2%)	80 (86.0%)	5.21	0.022*
	No (*n*, %)	12 (5.8%)	13 (14.0%)		
Always reading news about cardiovascular disease	Yes (*n*, %)	137 (66.2%)	60 (64.5%)	0.08	0.779
	No (*n*, %)	70 (33.8%)	33 (35.5%)		
Always attending to lectures about cardiovascular disease	Yes (*n*, %)	61 (29.5%)	40 (43.0%)	5.17	0.023*
	No (n, %)	146 (70.5%)	53 (57.0%)		
Always watching advertisements about diets and exercises	Yes (*n*, %)	135 (65.2%)	60 (64.5%)	0.01	0.906
	No (*n*, %)	72 (34.8%)	33 (35.5%)		

^*^P < 0.05; **P < 0.01;***P < *0.001*

#### Factors significantly related to ICVD in postmenopausal women: multivariate logistic regression analysis

The high-risk and low-risk groups as the dependent variables (0 for the low-risk group, 1 for the high-risk group), and the above described factors with statistical significance as independent variables were given the assignments (Table 3), and the Enter method was used to include variables. Then, the multi-factor logistic regression analysis was carried out (Table 4). The results suggested that monthly income, parity, whether to be exposed to second-hand smoke or third-hand smoke, whether to easily to buy healthy food, physical activities, depression, Type D personality, social supports and environment all were associated with the risk of ICVD in postmenopausal women (*P* < 0.05). OR > 1 indicated that the factors were positively correlated with ICVD, and the higher of the factor was, the higher the risk of ICVD was. OR < 1 indicated that the factors were negatively correlated with ICVD, and the higher of the factor was, the lower the risk of ICVD was. Our results suggested that easy access to healthy food (OR = 0.242), social support (OR = 0.861) and environmental factors (OR = 0.866) were the protective factors from ICVD. Parity (OR = 3.795), exposure to second-hand or third-hand smoke (OR = 2.886), depression (OR = 1.193), and Type D personality (OR = 1.148) were risk factors of ICVD. In addition, Table 4 shows that the OR values of monthly income and physical activities were close to 1, because they were the odds of ICVD per one unit of monthly income (¥) and per one score of physical activities. Therefore, the OR values of ICVD per 1000 units of monthly income (¥) and per 1000 scores of physical activities were 1.001^1000^ and 0.999^1000^, respectively, which suggested that monthly income was the risk factor of ICVD, and physical activities were the protective factor of ICVD.

**Table 3:** Assigning values to variables related to ischemic cerebrovascular disease in postmenopausal women

Variables	Assignments
Low- or high-risk cases	0 for low-risk cases; 1 for high-risk cases
Monthly income	Specific value
Gravidity	Specific value
Parity	Specific value
Physical activity	Specific value
Depression scoring	Specific value
Type D personality scoring	Specific value
Social support scoring	Specific value
Environment scoring	Specific value
Working conditions	1 for on job; 2 for unemployed
Whether to be exposed to second- or third-hand smoke	1 for yes; 2 for no
Whether to easily buy healthy food	1 for yes; 2 for no
Whether to attend lectures about cardiovascular disease	1 for yes; 2 for no

**Table 4:** Factors significantly related to ischemic cerebrovascular disease in postmenopausal women: multivariate logistic regression analysis

Factors	*β*	Wald ***χ***^***2***^ value	*OR*	*OR*(95%*CI*)	*P*
Monthly income (¥)	0.001	6.238	1.001	1.000–1.001	0.013*
Parity (times)	1.334	9.553	3.795	1.657–8.689	0.002**
Second-hand or third-hand smoke (yes versus no)	1.060	4.528	2.886	1.087–7.659	0.033*
Easy access to healthy food (yes versus no)	−1.417	3.875	0.242	0.059–0.994	0.049*
Physical activity (scores)	−0.001	21.875	0.999	0.998–0.999	< 0.001***
Depression (scores)	0.177	10.468	1.193	1.072–1.328	0.001**
Type D personality (scores)	0.138	8.013	1.148	1.044–1.264	0.005**
Social support (scores)	−0.149	10.996	0.861	0.789–0.941	0.001**
Environment (scores)	−0.143	13.033	0.866	0.801–0.937	< 0.001***

^*^P < 0.05; **P < 0.01;***P < *0.001*

## Discussion

Based on the SET, a multi-dimensional research and analysis was performed, and we found that the micro-level system, meso-level system and macro-level system in the social-ecological model all had an impact on the risk of ICVD in postmenopausal women.

### Analysis of factors affecting the risk of ICVD in postmenopausal women in the micro-level system of the social-ecological model

The multivariate logistic analysis revealed that the OR values of parity, exposure to second (third)-hand smoke, depression, and Type D personality were 3.795 (95% CI 1.657–8.689), 2.886 (95% CI 1.087–7.659), 1.193 (95% CI 1.072–1.328), and 1.148 (95% CI 1.044–1.264), respectively, which were all risk factors for ICVD in postmenopausal women.

Some studies suggested that parity was remarkably associated with the risk of carotid atherosclerotic plaque, and also associated with a decrease of HDL-C and an increase of the ratio of blood glucose/insulin [20, 21]. In addition, multiple parturition could increase the risk of metabolic syndrome in women, and thus, it might result in the increased risk of ICVD in postmenopausal women to a certain extent [22, 23]. Active or passive smoking, as a bad habit of life, has been found to be an independent risk factor for cardiovascular disease and also an important risk factor for adult death in our country [24–27]. The harmfulness of depression and Type D personality as negative psychological emotions have been confirmed by researches. A longitudinal study of middle-aged women implied that depression was an important risk factor for stroke [28]. In addition, it was also confirmed that depression, as a negative emotion, can increase the incidence and mortality of coronary heart disease and stroke [29, 30]. Type D personality is directly related to cardiovascular disease. Type D personality can cause the synergistic activation of the sympathetic adrenal system and hypothalamic–pituitary–adrenal axis to generate a strong salivary cortisol response which might be directly related to the occurrence of cardiovascular disease [31, 32]. In addition, Type D personality can activate the tumor necrosis factor alpha (TNF-alpha) and its receptors in patients with ischemic heart disease, causing the dysfunction of the human immune system [33, 34]. The OR value of physical activity was 0.999 (95% CI 0.998–0.999), and the OR value of monthly income was 1.001 (95% CI 1.000–1.001), indicating that these two factors had little association with the occurrence of ICVD. Although studies suggested that exercise was not only an effective and safe way to relieve menopausal symptoms in postmenopausal women [35], but also reduced the incidence of cardiovascular disease in postmenopausal women [36–38], the effect of physical activities on the occurrence of ICVD was not obvious in our study, which might result from the other factors that had significant effects on the occurrence of ICVD. Different studies have different opinions on the relationship between monthly income and the risk of cardiovascular disease. Some studies believed that economic income was not significantly associated with the risk of cardiovascular diseases [39], but some studies suggested the negative correlation between income and cardiovascular disease, and it would be reversed at a certain age, yet, with different opinions on the inflection point. One study indicated that the risk of cardiovascular disease was negatively correlated with economic income in people aged 50–64, while economic income in people older than 64 years did not have an influence on the risk of cardiovascular disease [40]. However, another study implied that the inflection point was 74 years old [41]. The results of a survey on dietary nutrients of residents in China suggested that with the increase of income, more and more people tend to have a poor dietary structure of high protein, high fat and low grain [42], which is likely to exacerbate the occurrence of cardiovascular disease. Therefore, the relationship between monthly income and cardiovascular disease is necessary to be further explored in the future.

### Analysis of factors affecting the risk of ICVD in postmenopausal women in the meso-level system of the social-ecological model

The OR value of social support in the meso-level system was 0.861 (95% CI 0.789–0.941), indicating that social support was a protective factor for ICVD in postmenopausal women. Social support includes not only subjective emotional supports, such as understanding and other pleasant positive emotions, but also objective and practical supports, such as material assistance, etc. What’s more, good utilization of social support and the correct way of seeking help constitute a complete social support system. Postmenopausal women with poor social support are more likely to be at high risk of ICVD. It have also been evidenced that lack of social support can aggravate the cardiovascular response of individuals under stress [43], while adequate social support has a buffer effect on the cardiovascular response caused by stress [44], which can only make a small increase in the blood pressure and heart rate of the population, thereby more easily returning to the normal level.

### Analysis of factors affecting the risk of ICVD in postmenopausal women in the macro-level system of the social-ecological model

The OR value of the environmental factor in the macro-level system was 0.866 (95% CI 0.801–0.937), implying that it was a protective factor. That exploration of the environment as a possible factor affecting the risk of cardiovascular disease was the first attempt in China, and our results suggested that good community living environment, including perfect living facilities, flat roads, good greening, good lighting conditions, and good and safe transportation, was a protective factor for postmenopausal women from ICVD, and might slow down the occurrence and development of ICVD to a certain extent, for which the reason might be that good community living environment could increase the convenience of going out, thereby increasing the level of physical activities of individuals [45–47]. This also provides a theoretical basis for the urban planning and environmental design in the future. Based on combining environmental hygiene with epidemiology, comprehensively considering the epidemiological characteristics of the disease is one of development trends in the future.

In addition, there are some limitations in our study. The sample size is small and only from one center. No long-term follow-up is performed. In the future, it is necessary to do the further study on this topic using multiple centers and large sample size, and do a long-term follow-up for those person whom finished the questionnaire.

In summary, the factors affecting the risk of ICVD in postmenopausal women include not only common micro-factors, but also psychological factors, social factors and environmental factors that are easily ignored. For the special group of postmenopausal women, in the future, in addition to prevention and control of the conventional risks, the conditions of their mentality and social support should be paid attention to, at the same time, and if they can, try to choose a good community environment to live in, in order to better reduce the incidence and mortality of ICVD in postmenopausal women.

## Data Availability

The datasets used and/or analyzed during the current study are available from the corresponding author on reasonable request.
